# *MdFRK2*-mediated sugar metabolism accelerates cellulose accumulation in apple and poplar

**DOI:** 10.1186/s13068-021-01989-9

**Published:** 2021-06-15

**Authors:** Jing Su, Chunxia Zhang, Lingcheng Zhu, Nanxiang Yang, Jingjing Yang, Baiquan Ma, Fengwang Ma, Mingjun Li

**Affiliations:** 1 State Key Laboratory of Crop Stress Biology for Arid Areas, Shaanxi Key Laboratory of Apple, College of Horticulture, Northwest A&F University, Yangling, 712100 Shaanxi China; 2 College of Forestry, Northwest A&F University, Yangling, 712100 Shaanxi China

**Keywords:** Cellulose, Hexose, Fructose, Fructokinase, UDPG, Primary phloem, *Malus* × *domestica*, *Populus*

## Abstract

**Background:**

Cellulose is not only a common component in vascular plants, but also has great economic benefits for paper, wood, and industrial products. In addition, its biosynthesis is highly regulated by carbohydrate metabolism and allocation in plant. MdFRK2, which encodes a key fructokinase (FRK) in apple, showed especially high affinity to fructose and regulated carbohydrate metabolism.

**Results:**

It was observed that overexpression of *MdFRK2* in apple decreased sucrose (Suc) and fructose (Fru) with augmented FRK activity in stems, and caused the alterations of many phenotypic traits that include increased cellulose content and an increase in thickness of the phloem region. To further investigate the involved mechanisms, we generated *FRK2*-OE poplar lines OE#1, OE#4 and OE#9 and discovered (1) that overexpression of *MdFRK2* resulted in the huge increased cellulose level by shifting the fructose 6-phosphate or glucose 6-phsophate towards UDPG formation, (2) a direct metabolic pathway for the biosynthesis of cellulose is that increased cleavage of Suc into UDP-glucose (UDPG) for cellulose synthesis via the increased sucrose synthase (SUSY) activity and transcript levels of *PtrSUSY1*, (3) that the increased FRK activity increases the sink strength overall so there is more carbohydrate available to fuel increased cambial activity and that resulted in more secondary phloem. These results demonstrated that *MdFRK2* overexpression would significantly changes the photosynthetic carbon flux from sucrose and hexose to UDPG for increased cellulose synthesis.

**Conclusions:**

The present data indicated that *MdFRK2* overexpression in apple and poplar changes the photosynthetic carbon flux from sucrose and hexose to UDPG for stem cellulose synthesis. A strategy is proposed to increase cellulose production by regulating sugar metabolism as a whole.

**Supplementary Information:**

The online version contains supplementary material available at 10.1186/s13068-021-01989-9.

## Background

Cell walls are one of the most important features of plant cells. About 70% of photosynthetic products are converted to polymers that accumulate in cell walls, and cell wall biomass is the most abundant renewable resource on earth [1]. Since the non-renewable nature of fossil energy has become a major challenge to sustainable human development, exploring how to convert cell wall biomass into usable energy is of great importance for human society.

The cell wall structure is complex and primarily consists of various polysaccharides, such as cellulose, hemicellulose, and pectin [2]. Cellulose, the most abundant renewable polymer in the biosphere [3], is already an important source of raw materials for textiles and paper products and has great potential for use in renewable biofuels or food production. Cellulose is a major component of the cell wall, where it exists in the form of microfibrils. Cellulose is composed of β-1,4-glucan chains linked by intermolecular hydrogen bonds and is made by cellulose synthase A (CESA) complexes (CSCs) at the plasma membrane that are believed to form sixfold-symmetrical rosettes [4]. *CesA* encodes a glycosyltransferase that belongs to the GT2 family and has been reported to have key roles in cellulose biosynthesis [5]. Indirect evidence suggested that *CESA-4* and *CESA-8* are associated with secondary wall cellulose synthesis in *Arabidopsis* [6]. Overexpression of a *CesA* mutant gene, *CesA7*^*fra5*^, in *Arabidopsis* led to a strong cellulose deficiency [7]. In addition, regulation of cellulose synthesis has a direct impact on plant growth and development. It is remarkable to note that reduced cellulose synthesis affects the softening of apples [8].

It is clear from studies of cellulose regulation that carbohydrate supply are essential for normal cellulose synthesis [9, 10]. Phloem is a transport system connecting source and sink, and it is required for the distribution of photosynthetic products (sugars) from source to sink organs [11]. In higher plants, photosynthetically active leaves export a large amount of carbon assimilates, primarily in the form of sucrose (Suc), via the phloem in support of sink development. Within the cytosol of sink cells, sucrose synthase (SUSY, EC 2.4.1.13) cleaves Suc to UDP-glucose (UDPG), which is the sole precursor for cellulose [12, 13]. SUSY activity has an important role in sink tissue metabolism and is a major area of interest within the field of cellulose biosynthesis [14, 15]. Another pathway of UDPG production from Suc is via neutral invertases (NINV, EC 3.2.1.26), hexose kinase enzymes, hexose phosphate interconversion enzymes and UDP-glucose pyrophosphorylase (UGPase, EC 2.7.7.9). Rende et al. [16] suggested that suppression of *NINV* resulted in a 38–55% reduction in NINV activity, a lower UDPG level, and a 9–13% decrease in cellulose content in poplar wood tissue.

Transport of Suc to the sink occurs via the phloem, and then in sink tissues NINV converts Suc into Glc and Fru, or SUSY converts Suc into UDPG and Fru [13, 17]. The resulting Glc and Fru can be phosphorylated by hexokinase (HxK, EC 2.7.1.1) and fructokinase (FRK, EC 2.7.1.4) to generate hexose phosphates, which are then converted to UDPG for cellulose biosynthesis [18]. FRK is the main fructose-phosphorylating enzymes *in planta* [19], and that *FRK2* orthologs play a vital role in controlling Fru utilization and carbon partition in hybrid aspen (*Populus tremula* × *tremuloides)* [20], apple (*Malus* × *domestica*.) [17], peach (*Prunus persica*) [21], and tomato (*Solanum lycopersicon* cv. MP1) [22]. Roach et al. showed that RNA interference of *FRK2* (RNAi-*FRK2*) in hybrid aspen decreased cell wall fiber thickness and the proportion of cellulose in the cell wall, implying that *FRK2* facilitates carbon allocation to cellulose in the wood [20]. Apple and other Rosaceae fruit trees transport more of their photosynthates as fructose, with more than 80% of the total carbon flux transporting as Fru into sink cells [23]. Our previous research demonstrated, compared with other plants, that *MdFRK2* is highly expressed in apple sink organs and that the MdFRK2 protein not only has a high-affinity for Fru but also a high enzymatic activity [17, 24], and overexpression *MdFRK2* in apple had a significant change in carbohydrate metabolism in the leaves [17]. Therefore, we inferred that augmented *MdFRK2* in plant sink cells might play a strong role in regulating sucrose metabolism and push more carbon forward for cellulose biosynthesis.

In this work, we observed that the transgenic apple with overexpressing *MdFRK2*, as our previous report [17], had significantly increased cellulose content and thickened the secondary phloem. To further investigate the involved mechanisms, we generated *MdFRK2*-OE poplar lines. The increased Fru phosphorylation in *MdFRK2*-transgenic poplar lines could promote the Suc cleavage via SUSY, and then generated more UDPG, which is the sole precursor for cellulose biosynthesis. *MdFRK2*-OE in poplar resulted in an increase in thickness of the secondary phloem region, but had no effects on stem height or diameter. We proposed a model that serves as a roadmap for future work to better understand the molecular network responsible for cellulose biosynthesis. These results demonstrated that *MdFRK2* overexpression in apple and poplar changes the photosynthetic carbon flux from Suc and hexose to UDPG for cellulose synthesis and will contribute to research on cellulose biosynthesis.

## Results

### Overexpression of *MdFRK2* increased cellulose level in transgenic apple

**Fig. 1:**
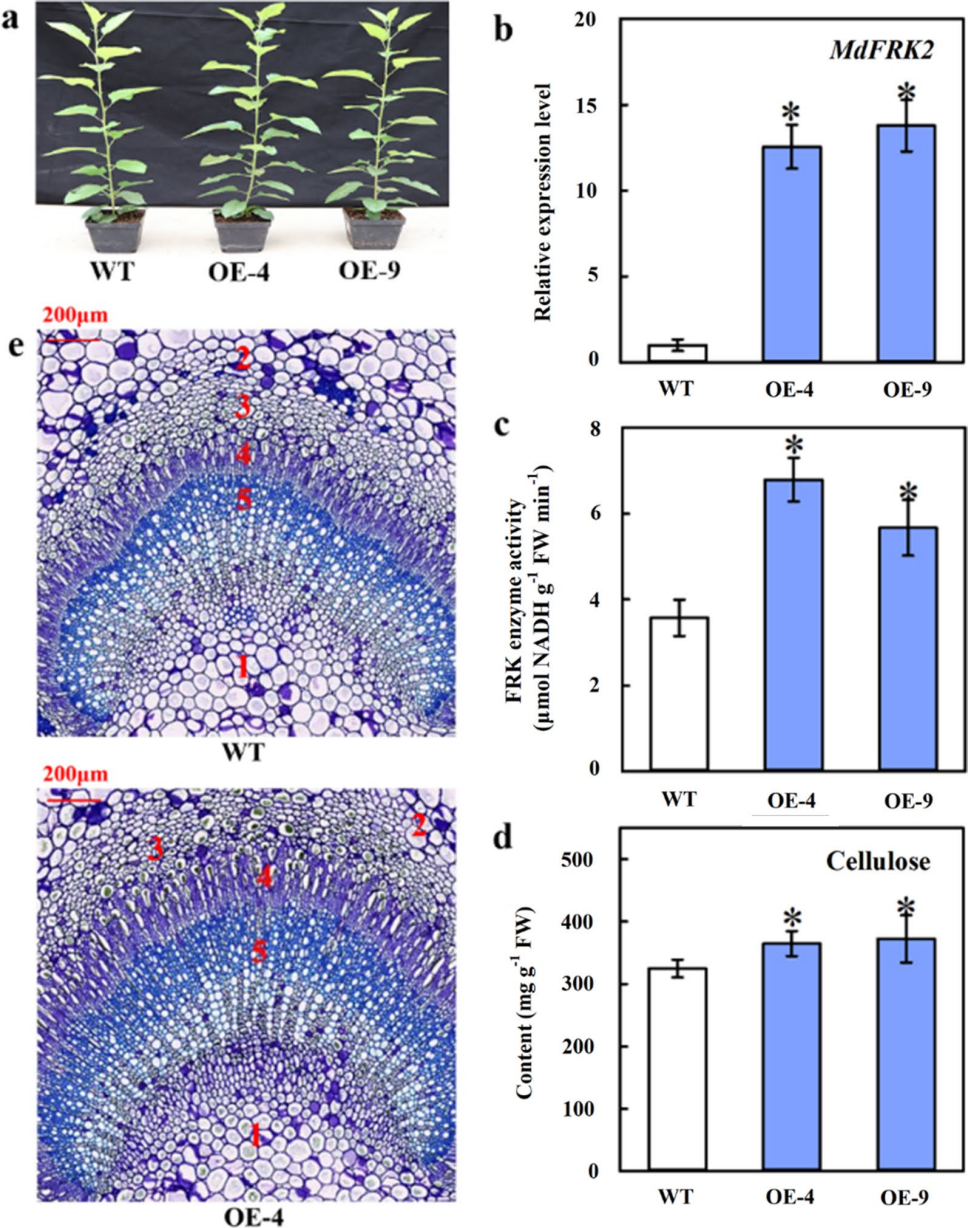
Molecular and microscopic characterization in stems of wild type (WT) and transgenic apple plants (OE-4 and OE-9). **a** Phenotypes of WT and transgenic lines (OE-4 and OE-9). **b, c** Quantitative RT-PCR of *MdFRK2* expression and FRK (fructokinase) activity in stems from WT, OE-4 and OE-9. In (**b**), the *MdActin* gene was used as internal control. **d** Content of cellulose in stems from WT and OE-4 and OE-9. Bars represent the mean value ± SE (*n* ≥ 3). The asterisk indicates significant differences at *P* ≤ 0.05. **e** Microstructure of petiole sections in WT and transgenic line OE-4. 1: pith; 2: parenchyma; 3: bast fiber; 4: phloem; 5: xylem. Scale bars = 200 μm

We generated *MdFRK2*-OE transgenic apple lines (OE-4 and OE-9) using the cauliflower mosaic virus 35S (CaMV 35S) promoter (Fig. 1a). Compared to wild type (WT), *MdFRK2* expression in the stems increased by 12.55- and 13.79-fold in lines OE-4 and OE-9, respectively (Fig. 1b). The activity of FRK was markedly increased in the OE lines (Fig. 1c). Cellulose contents of *MdFRK2*-OE transgenic apple stems were increased 0.12- and 0.15-fold, respectively, over WT (Fig. 1d). Light microscopy revealed that the *MdFRK2*-OE lines had significantly increased in thickness of the cambium and secondary phloem region compared to WT (Fig. 1e). These results showed that *MdFRK2* is a major player in cellulose synthesis in apple plants.

### Alteration of soluble carbohydrate concentration in *MdFRK2* transgenic apple

**Fig. 2:**
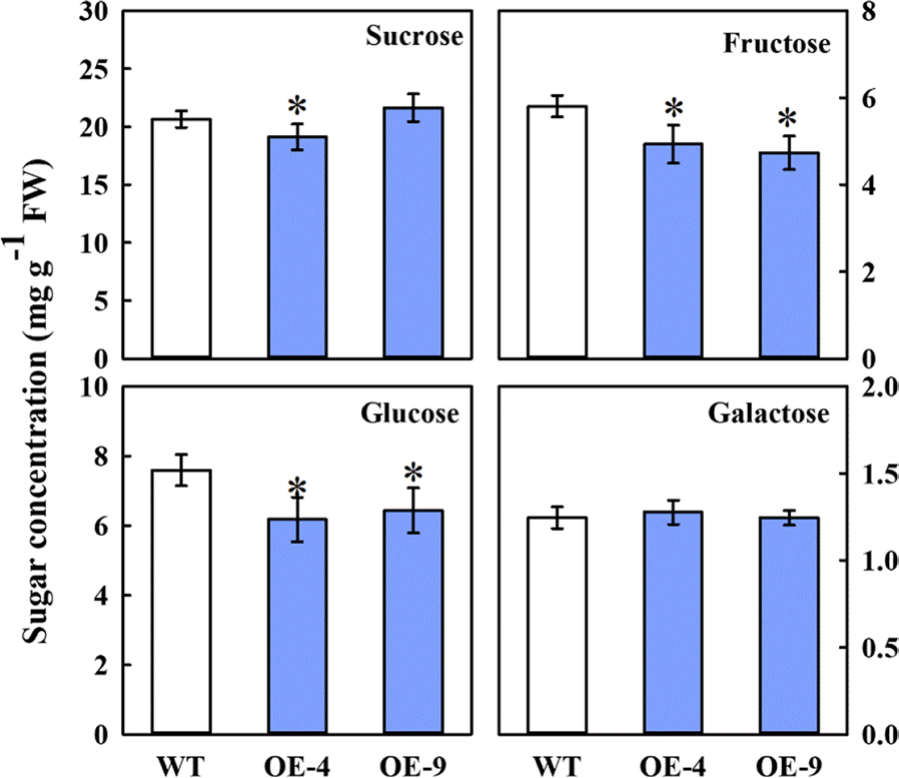
Sugar concentrations in the stems of the transgenic apple plants overexpressing *MdFRK2*. Sucrose, fructose, glucose and galactose concentrations were measured in wild-type WT and transgenic OE-4 and OE-9 lines. Bars represent the mean value ± SE (*n* ≥ 3). Asterisks indicate significant differences at *P* ≤ 0.05

To decipher how changes in the level of soluble carbohydrates affects cellulose synthesis with *MdFRK2* overexpression, the Fru concentrations in OE-4 and OE-9 transgenic lines were measured. Fru levels decreased by 15.00% in OE-4 and 18.28% in OE-9 (Fig. 2). It is worth mentioning here that the Suc concentration in the OE-4 transgenic line decreased significantly, by 7.50%, while Glc concentrations significantly decreased by 18.68% and 15.26%, respectively (Fig. 2). These results indicated that the links between *MdFRK2* and cellulose synthesis might be related to sugar metabolism in the sink.

### Heterologous expression of *MdFRK2* in poplar plants

**Fig. 3:**
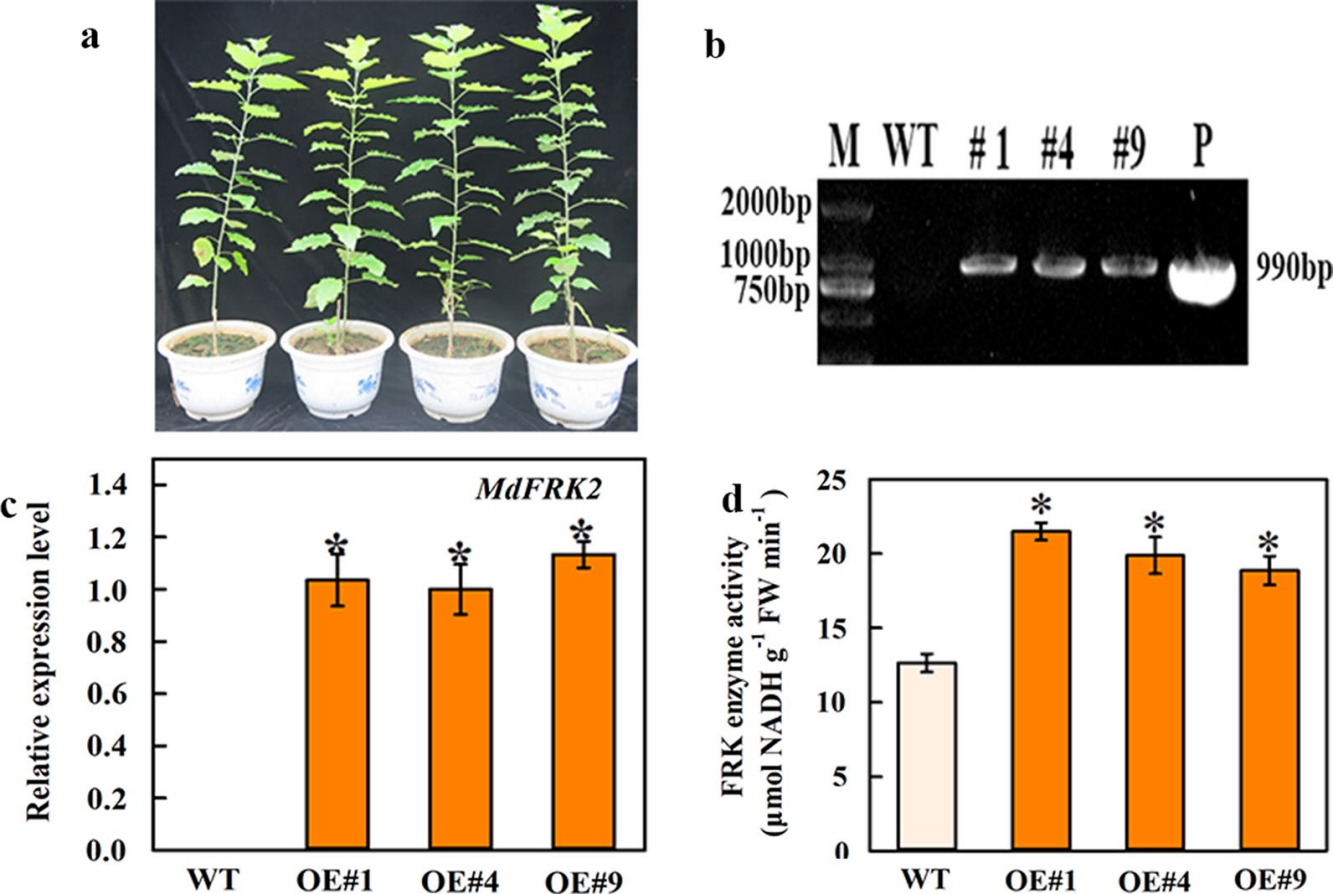
Characterization of wild type and transgenic poplar overexpressing apple *FRK2. a* Phenotype of wild type (WT, *Populus* clone 717) and transgenic lines (OE#1, OE#4 and OE#9). **b** DNA levels in the stems of WT and transgenic lines OE#1, OE#4 and OE#9. M, marker; P, plasmid. **c, d** Quantitative RT-PCR of *MdFRK2* expression and FRK (fructokinase) activity in stems from WT and transgenic lines OE#1, OE#4 and OE#9. In (**c**), the *PtrActin* gene was used as internal control. Bars represent the mean value ± SE (*n* ≥ 3). An asterisk indicates a significant difference at *P* ≤ 0.05

To further understand the cellulose synthesis-related functions of *MdFRK2* in stems, we heterologously expressed it in poplar (*Populus* clone 717) under the expression of CaMV promoter. Poplar is rich in cellulose and is an important source material for everyday products such as cloth, paper and biofuels [25]. Analysis of the DNA and mRNA levels revealed that three heterologous lines (OE#1, OE#4 and OE#9) (Fig. 3a) exhibited increases in *MdFRK2* transcript levels in stems relative to the levels in WT controls (Fig. 3b, c). All three lines also exhibited significantly increased enzyme activity. Interestingly, the FRK activity of mature leaves did not differ between the transgenic and wild-type plants (Additional file 1: Fig. S1). Lines OE#1, OE#4 and OE#9 displayed 0.70, 0.57 and 0.49-fold increases, respectively, in FRK activity in the stems, relative to the levels of the untransformed WT controls (Fig. 3d). Regardless of these observed differences, there were no significant changes in stem height and diameter in transgenic lines (Additional file 3: Table S1). These data showed that apple *FRK2* functioned very specifically in sugar metabolism in poplar.

## Heterologous expression of *MdFRK2* altered soluble sugar concentration of poplar plants

**Fig. 4:**
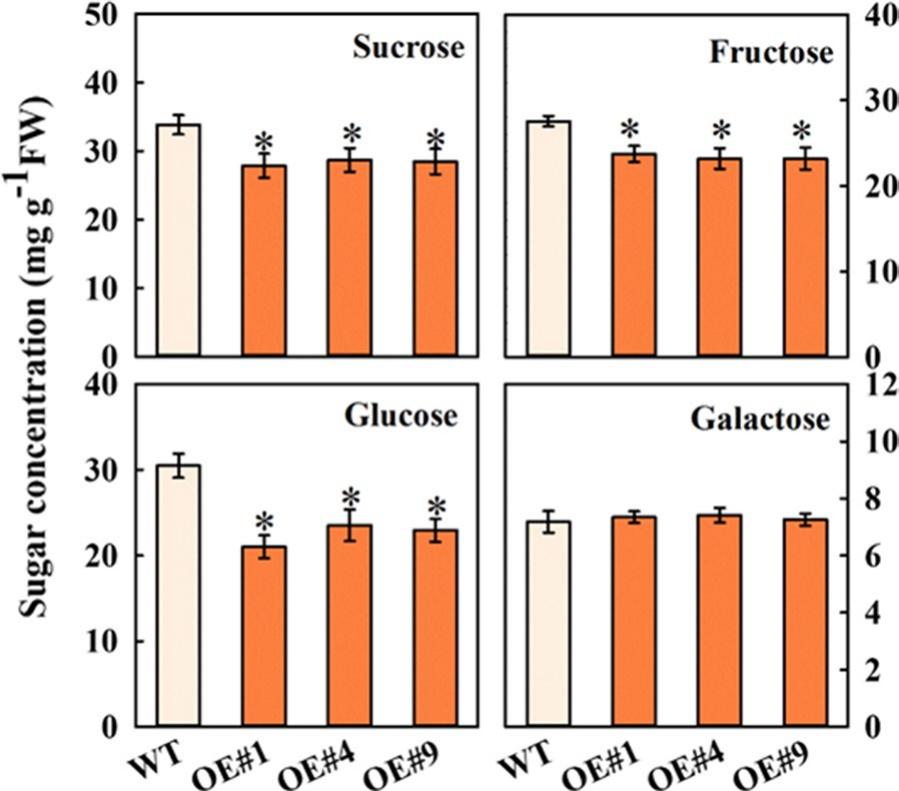
Sugar concentrations in the stems of transgenic poplar plants stems overexpressing *MdFRK2*. Sucrose, fructose, glucose and galactose concentrations were measured in wild type and transgenic OE#1, OE#4 and OE#9. Bars represent the mean value ± SE (*n* ≥ 3). An asterisk indicates significant differences at *P* ≤ 0.05

To examine the effect of overexpression of *MdFRK2* on carbohydrate metabolism in poplar, we determined sugar concentrations in transgenic lines using gas chromatography mass spectrometry GC/MS (Fig. 4). The high *MdFRK2* transcript levels in stems resulted in lower concentrations of Fru in the three transgenic lines, with levels reduced to 13.87% of the control level for OE#1, 15.76% for OE#4 and 15.80% for OE#9. The concentrations of Suc in these three transgenic lines were decreased by 17.65%, 15.16% and 15.84%, respectively. In the transgenic lines, the concentration of Glc was also decreased. These data demonstrated that *MdFRK2* modulate sucrose and hexose metabolism in a heterologous species.

### Carbohydrate metabolism pathway in stems of transgenic poplar

**Fig. 5:**
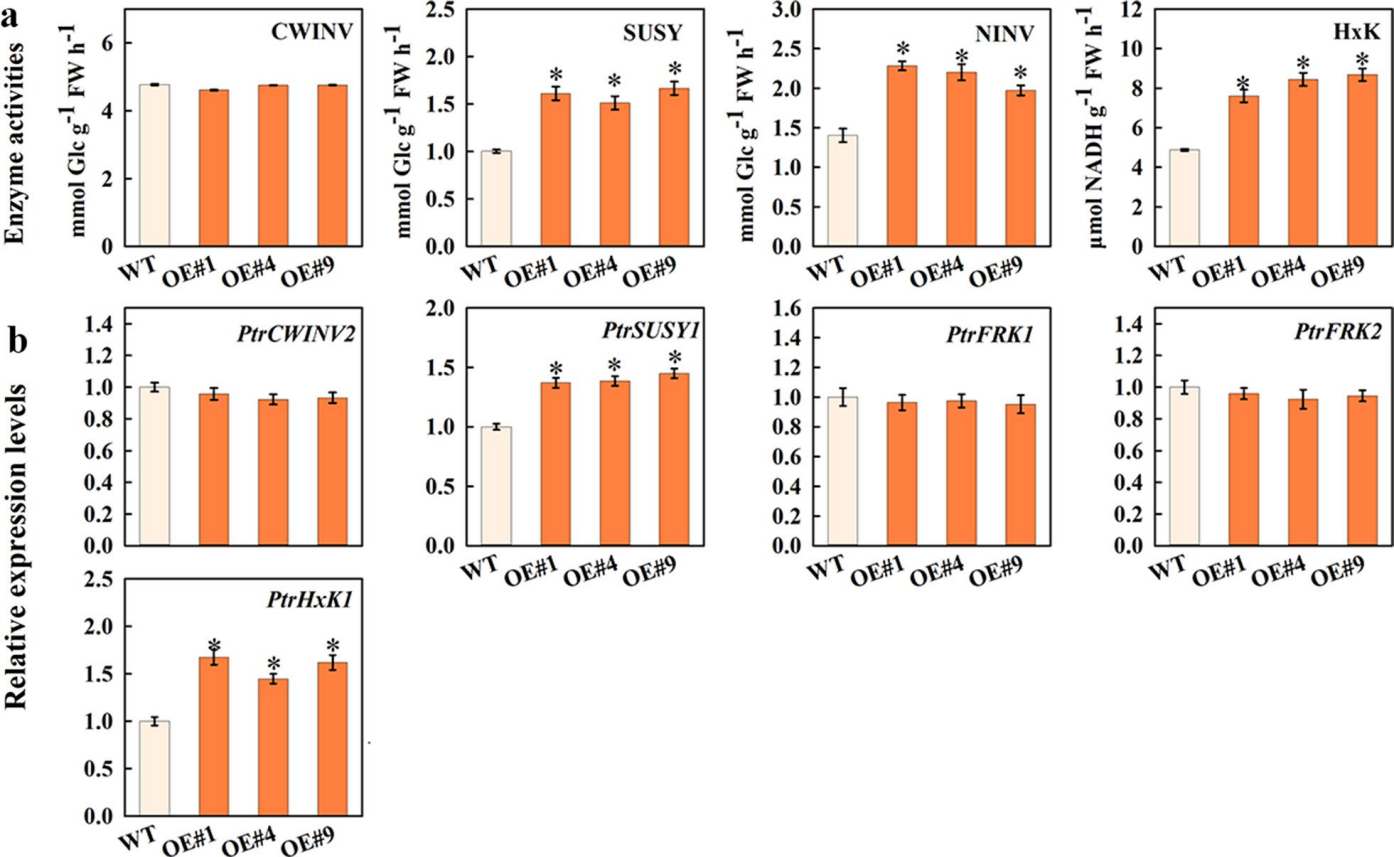
Enzyme activities and the expression of genes related to carbohydrate metabolism in the transgenic poplars (OE#1, OE#4 and OE#9) stems overexpressing *MdFRK2*. Activities of enzymes (**a**) and relative mRNA expression of genes (**b**) related to sugar metabolism in stems of wild type (WT, *Populus* clone 717) and transgenic lines (OE#1, OE#4 and OE#9). CWINV, cell wall invertase; NINV, neutral invertase; SUSY, sucrose synthase; HxK, hexokinase. Quantitative RT-PCR was performed with gene-specific primers using *PtrActin* as an internal control. Bars represent the mean value ± SE (*n* ≥ 3). An asterisk indicates significant differences at *P* ≤ 0.05

To determine why the sugar concentrations changed in the transgenic poplar lines expressing *MdFRK2*, enzyme activity and expression of genes related to sugar metabolism were assessed (Fig. 5a). The activity of cell wall invertase (CWINV) was not statistically different between the wild type and transgenic lines. However, the activities of neutral invertase (NINV) and sucrose synthase (SUSY), both of which are related to Suc breakdown, were significantly increased (Additional file 2: Figure S2). A similar pattern was observed for HxK and FRK activities. These results showed that the breakdown of Suc and Fru in sink cells is through the activities of SUSY and FRK, respectively. The changes in Suc and Fru were similar to the pattern of enzyme activities involved in Suc and Fru metabolism.

In addition, the transcript abundance of genes encoding these enzymes were investigated (Fig. 5b). Gene involved in Suc degradation (*PtrSUSY1*) was significantly upregulated in the transgenic poplar. In accord with the reduced Glc concentrations (Fig. 4), Transcripts for *PtrHxK1*, were increased. However, the expression level of *PtrCWINV2* was unchanged relative to control levels in transgenic lines. Taken together, these findings further suggested that the decreased Suc and Fru concentrations in *MdFRK2*-transgenic *Populus* were due to increased cleavage of Suc and Fru phosphorylation into hexose phosphates via the increased SUSY and FRK activities and transcript levels of *PtrSUSY1* and *MdFRK2*, respectively.

### Heterologous expression of ***MdFRK2*** accelerated UDPG accumulation of poplar plants

**Fig. 6:**
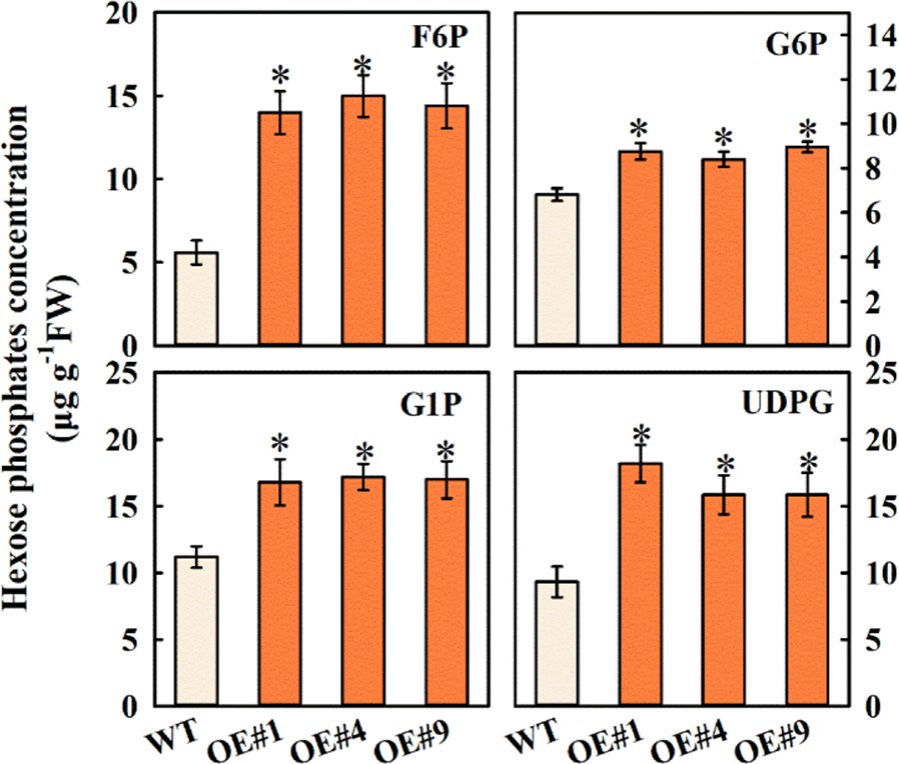
Hexose phosphate concentrations in stems of transgenic poplars (OE#1, OE#4 and OE#9) overexpressing *MdFRK2*. F6P, fructose 6-phosphate; G6P, glucose 6-phsophate; G1P, glucose 1-phsophate; UDPG, UDP-glucose. Bars represent the mean value ± SE (*n* ≥ 3). An asterisk indicates significant differences at P ≤ 0.05

SUSY directly produces UDPG, which is the substrate for cellulose synthesis in sink organs. To confirm that alteration of UDPG in the transgenic lines was caused by increased SUSY or FRK activity, the UDPG concentration was detected (Fig. 6). In poplar overexpressing *MdFRK2*, the concentrations of UDPG were significantly increased, by 0.95-, 0.70- and 0.69-fold, respectively. Furthermore, the levels of fructose 6-phosphate (F6P), glucose 6-phsophate (G6P) and glucose 1-phsophate (G1P) were increased greatly in all three OE lines relative to the levels in WT. These results suggested that *MdFRK2* overexpression could accelerate UDPG accumulation and that the capacity for cellulose synthesis via UDPG would be increased.

### Heterologous expression of MdFRK2 accelerated cellulose accumulation in Populus plants

**Fig. 7:**
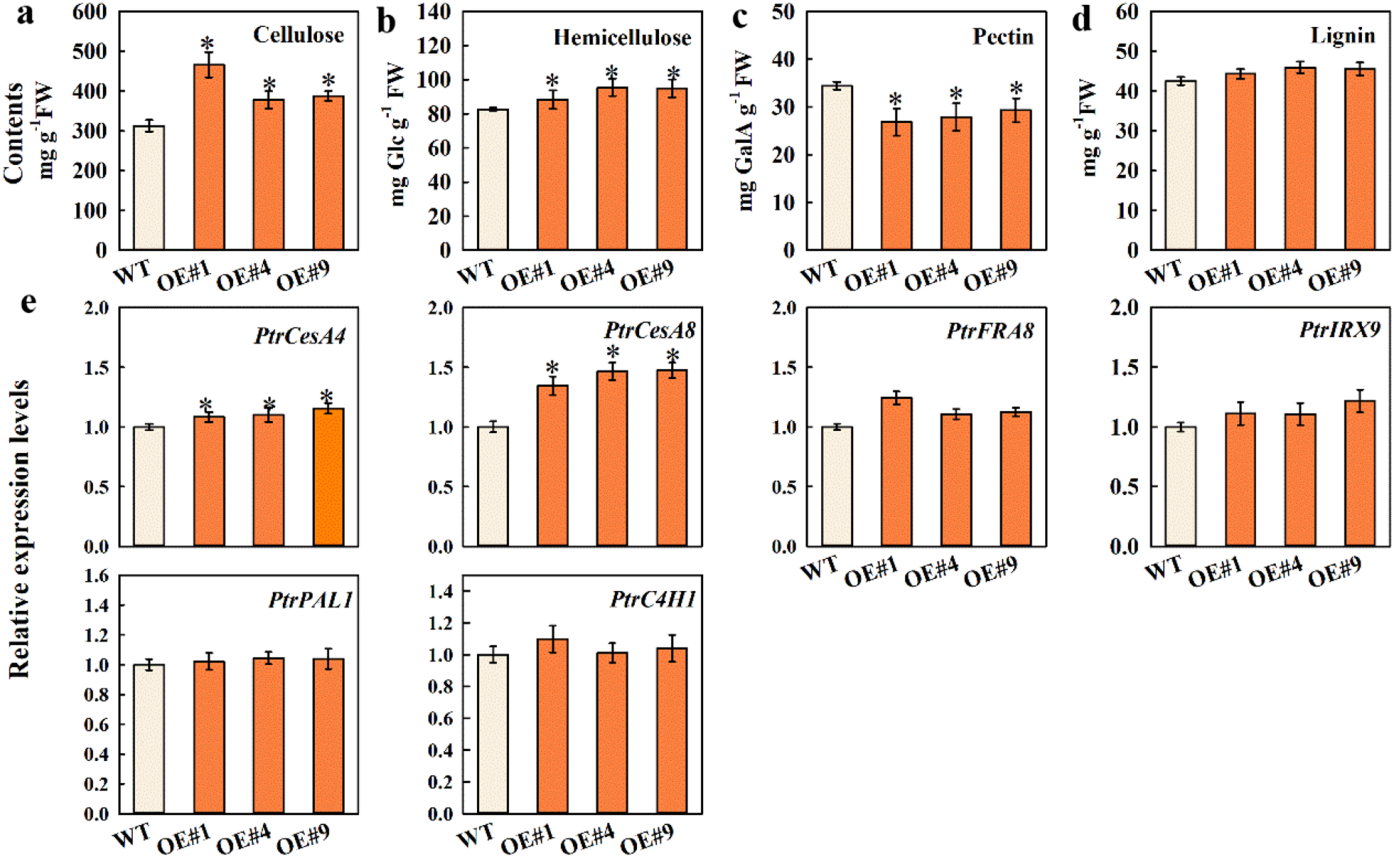
Contents of cellulose, hemicellulose, pectin, lignin and the expression of genes that are involved in the biosynthesis of cellulose (*PtrCESA4*, and *PtrCESA8*), hemicellulose (*PtrFRA8*, and *PtrIRX9*), and lignin (*PtrPAL1*, and *PtrC4H1*) in stems of the transgenic poplar lines (OE#1, OE#4 and OE#9) overexpressing *MdFRK2*. Contents of cellulose, hemicellulose, pectin, lignin (**a**–**d**) and relative mRNA expression of genes (**e**) that are involved in the biosynthesis of cellulose (*PtrCESA4*, and *PtrCESA8*), hemicellulose (*PtrFRA8*, and *PtrIRX9*), and lignin (*PtrPAL1*, and *PtrC4H1*) in stems of wild type (WT, *Populus* clone 717) and transgenic lines (OE#1, OE#4 and OE#9). Quantitative RT-PCR was performed with gene-specific primers using *PtrActin* as an internal control. Bars represent the mean value ± SE (*n* ≥ 3). An asterisk indicates significant differences at *P* ≤ 0.05

After seeing increased UDPG levels in stems of these three transgenic lines with up-regulated *MdFRK2* expression, the cellulose content of all transgenic lines were measured (Fig. 7a). The contents of cellulose increased 0.49-, 0.21- and 0.24-fold in the three transgenic lines as compared with WT, respectively. Accordingly, the three transgenic poplar lines contained increased hemicellulose contents, by 6.90% in OE#1, 15.64% in OE#4 and 14.84% in OE#9 (Fig. 7b). The pectin contents in the transgenic poplar stems decreased 0.22-, 0.19- and 0.15-fold compared to WT (Fig. 7c), whereas the lignin content showed no significant changes (Fig. 7d).

To further identify key genes contributing to changes in cellulose levels in the transgenic lines, the expression levels of genes related to these traits were measured using qRT-PCR. The results suggested that the expression levels of genes involved in the biosynthesis of cellulose (*PtrCesA4* and *PtrCesA8*), were highly up-regulated in OE#1, OE#4 and OE#9 transgenic lines compared to in wild type (Fig. 7e). However, the expression levels of hemicellulose (*PtrFRA8* and *PtrIRX9*) and lignin biosynthetic genes (Ptr*PAL1* and Ptr*C4H1*) did not obviously differ. These results demonstrated that cellulose is a major sink for *FRK2*-metabolized carbon.

### Heterologous expression of MdFRK2 changed secondary phloem in Populus plants

**Fig. 8:**
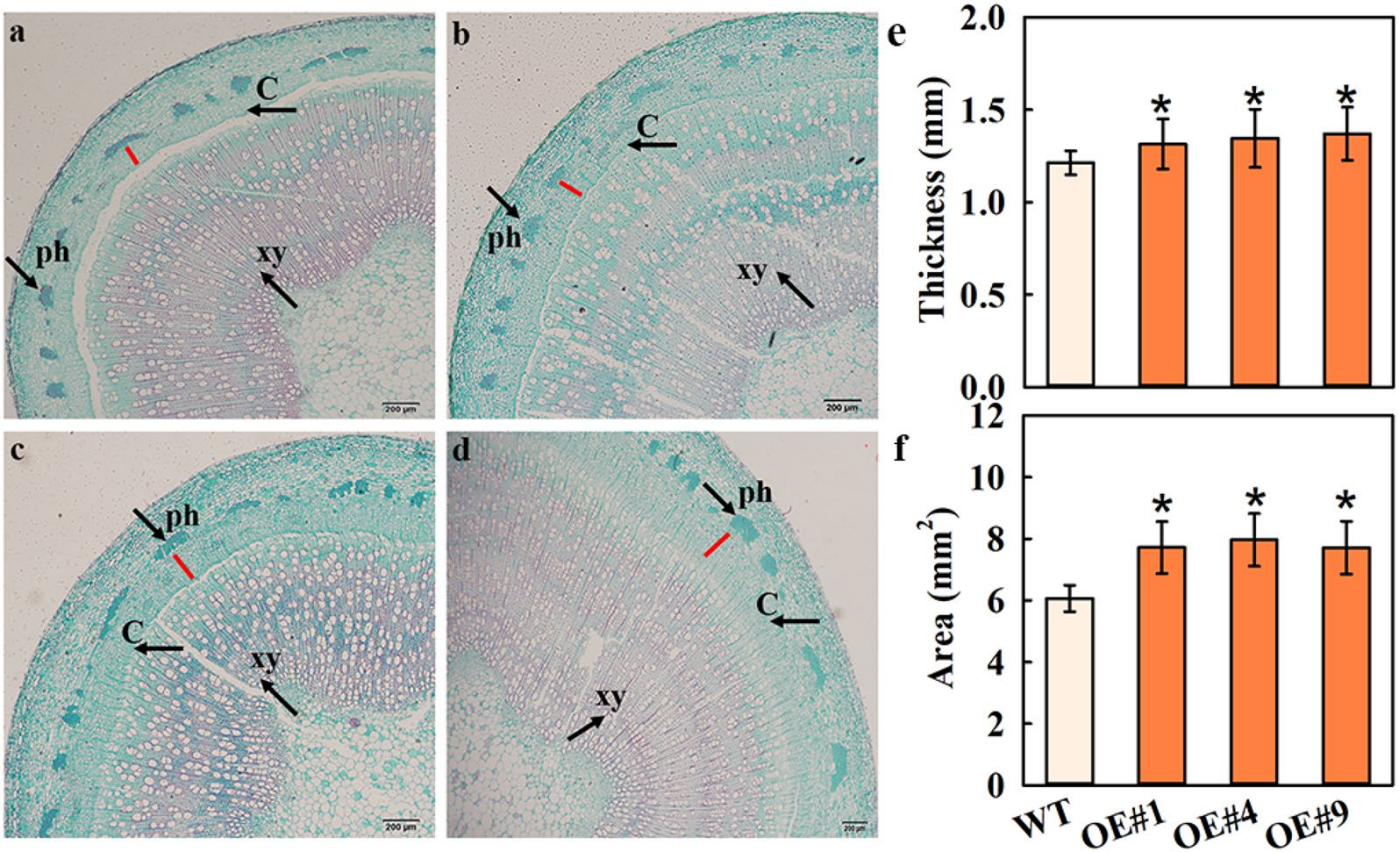
Secondary phloem of the transgenic poplar lines (OE#1, OE#4 and OE#9) overexpressing *MdFRK2*. **a–d** Cellulose (green color) in stem sections stained with safranin and fast green. Scale bars = 200 μm. **a** Wild type (WT, *Populus* clone 717); **b**: transgenic line OE#1; **c**: transgenic line OE#4; **d**: transgenic line OE#9. ph: phloem (black arrow); xy: xylem (black arrow); C: cambium (black arrow); red lines: the cambium and secondary phloem region. An increase in thickness **e** and area **f** of the secondary phloem region in transgenic lines (OE#1, OE#4 and OE#9) in comparison with WT. Bars represent the mean value ± SE (*n* ≥ 3). The asterisk indicates a significant difference at *P* ≤ 0.05

As reported previously [26], the cambium, is a meristem between xylem and phloem, produces secondary phloem. To test whether the increased cellulose level altered the secondary phloem of transgenic lines, safranin and fast green were used to stain cellulose and lignin, respectively. Examination by light microscopy revealed that OE#1, OE#4 and OE#9 transgenic lines had significantly increased in thickness of the   secondary phloem region (Fig. 8a–d), which was increased by 8.33%, 10.96% and 12.94% compared to wild type, respectively (Fig. 8e). These results indicated that increased FRK activity increases the sink strength overall so there is more carbohydrate available to fuel increased cambial activity and that resulted in more secondary phloem.

## Discussion

### *MdFRK2* is essential for cellulose biosynthesis in apple plants

Photosynthetically active leaves produce many forms of carbohydrates, such as Suc, which serve as energy and carbon sources for cellulose synthesis in the sink of most plant species. There have been many reports suggested that accelerated Suc decomposition leads to increased cellulose content in *SUSY* overexpression plants [27–29]. More recently, FRK2 is generally considered a main regulator that functions in carbohydrate metabolism in the leaves [17]. However, it remains unknown how *MdFRK2* plays a role in regulating sugar metabolism for cellulose biosynthesis.

In this report, two *MdFRK2*-OE transgenic apple plants, OE-4 and OE-9, were observed (Fig. 1a). The significant increases in *MdFRK2* transcript levels and FRK enzyme activity in stems suggest that *MdFRK2* may have a special function in stem development. Similarly, the influence of the *FRK2* gene on cell-wall biosynthesis was reported in aspen, suggesting that the reduction in *FRK2* activity in *FRK2*-RNAi hybrid aspen primarily affected cellulose [20]. Thus, we hypothesized that apple *FRK2* plays direct roles in cellulose synthesis. As we had expected, elevated *MdFRK2* transcript levels significantly increased the cellulose content and resulted in an increase in thickness of the cambium and secondary phloem region in the OE lines. Our data demonstrated that *MdFRK2* aids cellulose synthesis in apple plants. In addition, the previous studies have reported that hybrid aspen *FRK2* could effectively control carbon flux into cell walls for cellulose biosynthesis [20]. In the present study, overexpression of *MdFRK2* resulted in significantly lower concentrations of soluble sugar in the *MdFRK2*-OE apple lines (Fig. 2), which is similar to the results from previous studies of apple and aspen as discussed. In light of these results, we speculated that the links between *MdFRK2* and cellulose synthesis might be related to sugar metabolism in the sink.

### Cellulose is a major sink for FRK2-metabolized carbon

Poplar is more richness in cellulose than apple, and poplar is an important source for the raw materials used in everyday products such as cloth, paper and biofuels [30]. To study the functions of *MdFRK2* from apple, we heterologously expressed *MdFRK2* in *Populus* and obtained three lines overexpressing the fructokinase gene (Fig. 3a, b). As expected, the mRNA levels and enzyme activity in stems increased significantly (Fig. 3c, d, Additional file 1: Fig. S1) due to *MdFRK2* overexpression. This result was expected, as it was previously reported that overexpression of *MdFRK2* in apple significantly increased *MdFRK2* transcription and FRK enzyme activity [17]. Surprisingly, this increased FRK2 activity resulted in altered cell wall cellulose content, with increases of 21% to 24% over control levels (Fig. 7a), without affecting plant growth (Additional file 3: Table S1). Taken together, these results are consistent with previous reports that the function of apple *FRK2* is very specific for cellulose biosynthesis in poplar.

It is particularly notable that overexpression of *MdFRK2* in transgenic poplars caused significant decreases in soluble sugar and increases in hexose-phosphates levels, especially UDPG (Figs. 4, 6). Moreover, the key enzymes and the expression levels of main genes related to soluble sugar metabolism also showed a significant increase (Fig. 5). Thus, we suggest that cellulose synthesis might be related to modulation of Suc and hexose metabolism via *MdFRK2*. Here we present lines of direct evidence to explain which mechanisms might be involved in this. First, cellulose is generated from the precursor UDP-glucose (UDPG), which can be derived from the cleavage of Suc by SUSY to directly yield UDPG and Fru [31]. In this context, our data are in line with previous reports that showed that the increased cellulose in *MdFRK2*-transgenic poplars was due to increased cleavage of Suc into UDPG via the increased SUSY activity and transcript levels of *PtrSUSY1* (Figs. 4, 5 and 6, Additional file 2: Fig. S2). Similar results have been found in cotton [15], hybrid aspen [20], and wheat (*Triticum aestivum* L.) [32], with overexpression of SUSY being associated with increased cellulose synthesis. These data showed a direct metabolic pathway for the biosynthesis of UDPG to cellulose.

Second, the resulting Fru and Glc can be readily phosphorylated by the increased activities of FRK and HxK enzymes to generate hexose-phosphates that are then converted to UDPG for cellulose synthesis. Here, *MdFRK2*-overexpressing transgenic poplar showed that transcripts of other FRKs were not influenced (Fig. 5b), suggesting that the increased FRK activity is, therefore, due to an increase in *MdFRK2* activity. This increase correlated with decreased Fru and increased F6P, UDPG and cellulose levels (Figs. 4, 6, 7a). It is, therefore, plausible that high FRK activity and the resulting decrease in Fru levels could lead to an increase in cellulose by shifting the F6P towards UDPG formation. A similar conclusion comes from a study of *FRK2*-RNAi in hybrid aspen [20]. Furthermore, it is important to note that G6P, a hexose-phosphate, is an important intermediate in cellulose synthesis [33]. In *MdFRK2*-transgenic poplar plants, the resulting Glc in carbon metabolism will be phosphorylated by the increased HxK enzyme activity (correlated to increased transcript levels of *PtrHxK1*) to produce G6P (Fig. 4, 5 and 6). G6P has three possible fates, namely, (1) entering glycolysis/TCA cycle, (2) being used for starch synthesis, (3) being converted to UDPG for cellulose synthesis. These findings provide novel insights into the relationship between *MdFRK2* and cellulose synthesis [13, 34].

Besides cellulose, hemicelluloses and pectins influence both crystallinity and network connectivity of cellulose microfibrils in cell walls [5]. In this study, our data showed a significant increase in hemicellulose content in transgenic poplar and a reduction in the pectin concentration when compared with WT (Fig. 7b, c). The data support the idea that increased SUSY activity in transgenic tobacco could distribute more carbon to hemicellulose synthesis, but less to pectins, as previously described [31]. A possible explanation for this might be that the increased *MdFRK2* activity provided more carbohydrate substrates to hemicellulose, which is in line with the described mechanism that links *MdFRK2* with regulated cellulose or hemicellulose production and might be related to its ability to regulate carbon distribution. It is interesting to note that the expression of genes (*PtrFRA8* and *PtrIRX9*) related to hemicellulose [35] were unchanged in relative to control levels in transgenic poplars (Fig. 7e).

### Potential basis for cellulose biosynthesis in apple and poplar

A multiple transmembrane spanning protein, CESA, directly influences cellulose content. UDPG is the substrate of the CESAs [36]. Indirect evidence suggests that *CESA4* and *CESA8* seem to be absolutely necessary for secondary wall cellulose synthesis [6]. In this study, the expression of genes involved in cellulose biosynthesis (*PtrCesA4* and *PtrCesA8*) were significantly increased in *MdFRK2*-overexpressing transgenic poplars (Fig. 7e). Based on these data, we believe that there is a positive correlation between cellulose synthase and *CESA* gene expression. The importance of plant *FRK2* for the development of vascular tissues has been documented in sinks in hybrid aspen [20] and tomato [37]. In our report, overexpression of *MdFRK2* resulted in augmented cellulose levels in transgenic poplar and an increase in thickness of the secondary phloem region, which increased 10.96% to 12.94% compared to wild type (Figs. 7a, 8a–e). Together with the increased cellulose levels, these results supported the view that with increased FRK activity increases the sink strength overall so there is more carbohydrate available to fuel increased cambial activity and that resulted in more secondary phloem, suggesting that *MdFRK2* plays a general role in carbon partitioning to UDPG for cellulose synthesis.

## Conclusion

**Fig. 9:**
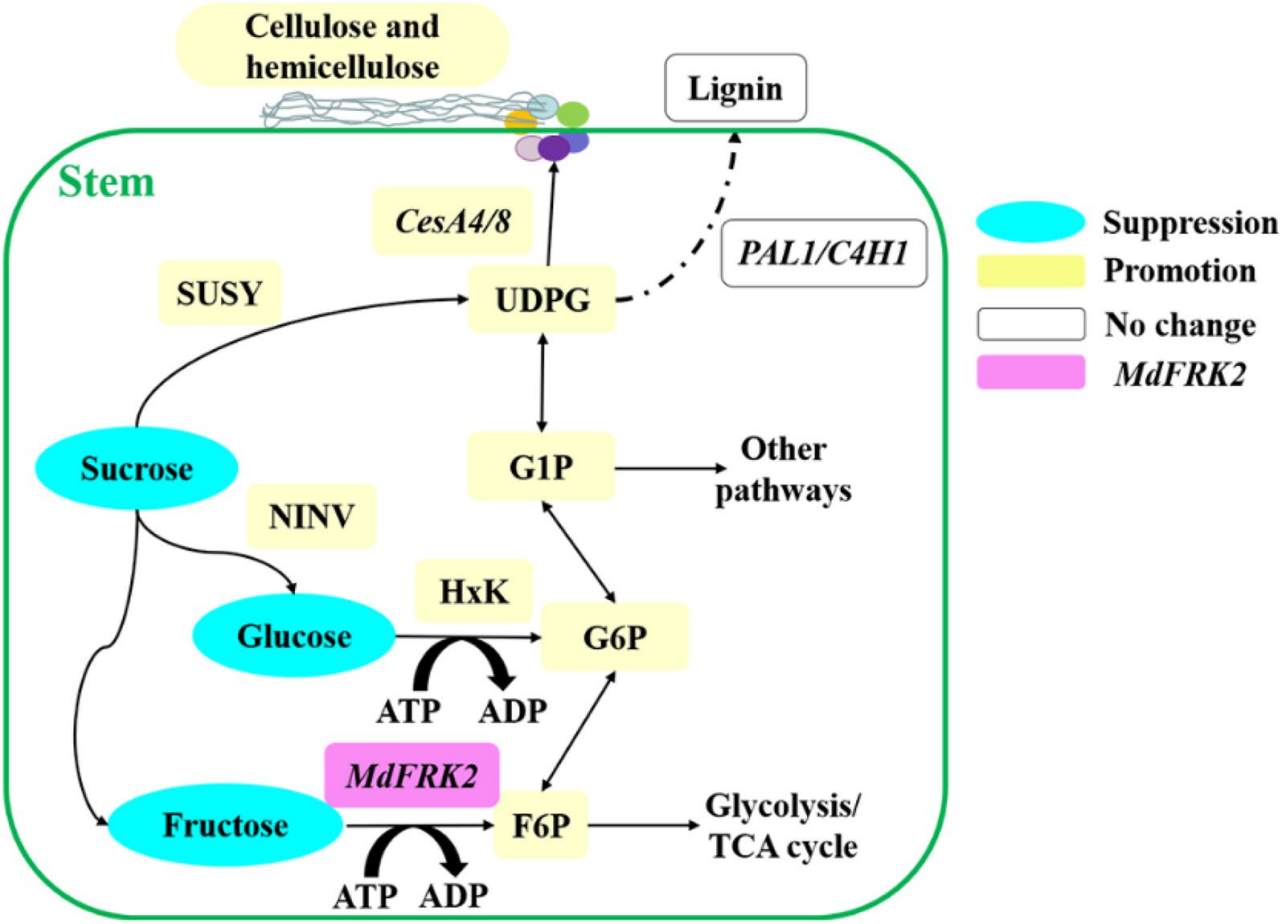
Proposed model of *MdFRK2*-regulated cellulose synthesis in apple and poplar. In stems, a direct metabolic pathway for the biosynthesis of cellulose is through the increased cleavage of Suc into UDPG via increased SUSY activity, which is an alternative pathway for UDPG production from Suc via NINV. One observation of this study is that overexpression of *MdFRK2* resulted in increased cellulose content by shifting F6P or G6P towards UDPG formation. With increased FRK activity increases the sink strength overall so there is more carbohydrate available to fuel increased cambial activity and that resulted in more secondary phloem. Blue boxes indicate a reduction in soluble sugar concentrations. Yellow boxes indicate increases in soluble sugar or hexose phosphate concentrations, enzyme activity or relative mRNA expression of genes. White boxes, no change in relative mRNA expression of genes that are involved in the biosynthesis of lignin (*PAL1*, and *C4H1*). Purple boxes indicate the relative mRNA expression of *MdFRK2*

The results reported here suggest that (1) overexpression of *MdFRK2* could increase carbon flux to UDPG for cellulose biosynthesis, which can then be used for phloem development in apple and poplar plants; (2) a direct metabolic pathway for the biosynthesis of UDPG is through increased cleavage of Suc into UDPG via the increased SUSY activity and transcript levels of *SUSY1*; (3) an alternative pathway of UDPG production from Suc via NINV; and (4) overexpression of *MdFRK2* results in increased cellulose content by shifting F6P or G6P towards UDPG formation (Fig. 9). These results provide insights into the role of *MdFRK2* in controlling cellulose or hemicellulose production that might be related to its ability to regulate sugar metabolism.

## Materials and methods

### Plant materials

Tissue-cultured WT and and *MdFRK2*-transformed ‘GL-3’ apple plantlets were grown on Murashige and Skoog (MS) medium supplemented with 0.2 mg L^−1^ IAA, 0.3 mg L^−1^ 6-BA and 25 mg · L − 1 kanamycin for 4 weeks. They were then rooted in MS medium (MS + 0.5 mg IBA and 0.5 mg IAA) for 2 months.

Untransformed poplar WT (*Populus* clone 717) and *MdFRK2*-transformed ‘*Populus* clone 717’ poplar plantlets were grown on MS medium containing 0.25 g L^−1^ MES, 0.1 g L^−1^ Inositol and 0.3 g L^−1^ l-glutamine for 2 months.

After rooting, all genotypes were transferred to a culture room at Northwest A&F University, Yangling, Shaanxi, China, under conditions previously reported by Wang et al. [38]. After these plants had grown for 2 months, stems were collected and immediately frozen in liquid nitrogen and stored at −80 ºC.

### Cloning of *MdFRK2*

The *MdFRK2* sequence (MD04G1042400) was retrieved from the Malus Genome Database (http://www.rosaceae.org). Specific primers were designed for gene cloning (Additional file 2: Table S2). Total RNA was extracted from young fruits of ‘Gala’ apple, and cDNA was synthesized using PrimeScript™ II Reverse Transcriptase (Takara, Dalian, China).

### Vector constructs and *Populus* transformation

The coding region of *MdFRK2* was cloned into the gateway vector PGWB402 with the CaMV 35S promoter. The recombinant plasmid was transformed into *Agrobacterium tumefaciens* strain EHA105 for transformation. The transformation of *Populus* was done according to the procedure of Dai et al. [39]. Afterward, Popular plants were grown at 24 °C under a 15-h photoperiod supplemented with fluorescent light at 60 μmol m^−2^ s^−1^.

Transgenic plants were screen by kanamycin resistance and PCR analysis of extracted DNA. From the four overexpression transgenic lines obtained, we selected three for further analysis. Untransformed WT served as the control plants.

### Sugar concentration measurement

As recently described [40], soluble sugars and hexose phosphates were extracted and then derivatized with methoxyamine hydrochloride and Nmethyl-N-trimethylsilyl-trifluoroacetamide. After derivatization, the metabolites were analyzed using a Shimadzu GC/MS-2010SE (Shimadzu Corporation, Tokyo, Japan).

### Enzyme assay and expression analysis

CWINV, SUSY, NINV, FRK, and HxK in steam samples were extracted as described [13]. Soluble proteins were measured using Coomassie blue, and enzyme activities were expressed on a protein basis.

Total RNA from frozen tissues was extracted with RNAprep plant kit (CWBIO, Beijing, China), and cDNA was synthesized using PrimeScript™ II Reverse Transcriptase (Takara, Dalian, China).

Gene-specific primers were designed in NCBI and used for qRT-PCR. The analysis of PCR products was done according to procedure [37]. Data were analyzed using the ΔΔCT method. Primers used in this study are listed in Additional file 4: Table S2.

### Histological analysis

Stems and petiole were cut into 0.5-cm segments and fixed in FAA stationary liquid at 4 °C for 3 days, drawn under a vacuum for 1 h, dehydrated in a graded ethanol series (2-h each), cleared in dimethylbenzene: ethanol (50: 50, v/v), and then cleared in 100% dimethylbenzene twice, for 2 h each time. Tissues were embedded in dimethylbenzene: wax (50: 50, v/v) overnight at 60 °C for 6 h, and then embedded in paraffin. The embedded tissues were sectioned to 4-μm thickness for staining using a Leica RM 2235 microtome (Leica) and adhered to microscope slides (Thermo Fisher) at 42 °C for 15 min. Afterward, some sections were stained with safranin and fast green for lignified cell wall and cell wall of phloem observation, which, respectively, take on red and green color under a light microscope. Finally, imaging was performed with an Olympus BX51 light microscope. The vascular bundle areas of three different tissue samples were quantified in a randomly selected area using Image J software (http://rsbweb.nih.gov/ij/).

### Cellulose, hemicellulose, pectin and lignin content analyses

Cellulose was extracted according to the procedure used by Sun et al. [41] with some changes. Samples were analyzed for hemicellulose and pectin as previously reported [42]. Briefly, the stems were ground in liquid nitrogen, followed by one extraction with 70% ethanol and three extractions with chloroform: methanol (1:1, v/v). Aliquots (0.50 g and 0.10 g) of alcohol-insoluble residue were prepared for determination of cell wall components. First, the 0.50-g aliquot was homogenized in 90% DMSO, after which it was resuspended in 50 mM CDTA, 50 mM Na_2_CO_3_ and 24% KOH and centrifuged at 8000 r/min for 5 min; the residue was washed three times with 100% acetone and then freeze-dried; the solids were considered as cellulose. The 0.10-g aliquot was treated with 50 mL of 72% sulfuric acid at room temperature for 1 h. Sulfuric acid was then diluted to 4%, and the mixture was heated at 121 °C for 1 h. Released monosaccharides were analyzed using an ICS-3000 HPLC system (Thermo Fisher Scientific). Hemicellulose was calculated using the amount of Glc released by 4% sulfuric acid. Pectin concentration was calculated based on the amount of galacturonic acid (GalA). The lignin concentration was measured according to our previous method [43], in which extracted ground stem tissue (0.1 g) was treated with 3 ml of 72% H_2_SO_4_ and stirred every 10 min for 2 h. The sample was then diluted with 112 ml of deionized water and autoclaved at 121 °C for 1 h. The acid-soluble lignin component was determined at 205 nm by spectrophotometry.

### Statistical analysis

SPSS Statistics 21 (SPSS, Inc., Chicago, IL, United States) was used to analyze all data in this study. Data was graphed with Sigma Plot 12.0 software. Data were analyzed using an independent *t* test, with a significance level of *P* ≤ 0.05. Values are presented as the mean ± standard error (SE) in at least biological triplicate for each measurement.

## Supplementary Information

**Additional file 1: Fig. S1.** FRK enzyme activity in the transgenic poplars (OE#1, OE#4 and OE#9) leaves overexpressing *MdFRK2*.

**Additional file 2: Fig. S2.** SUSY activity in stems of transgenic poplars (OE#1, OE#4 and OE#9) overexpressing *MdFRK2*.

**Additional file 3: Table S1.** Phenotypic characteristics of the transgenic poplars (OE#1, OE#4 and OE#9) overexpressing *MdFRK2*.

**Additional file 4: Table S2.** Primers used in this study.

## Data Availability

The data sets supporting the conclusions of this article are included within the article and its additional files.

## Abbreviations

Suc: Sucrose
Glc: Glucose
Fru: Fructose
OE: Overexpression
UDPG: UDP-glucose
SUSY: Sucrose synthase
F6P: Fructose 6-phosphate
G6P: Glucose 6-phsophate
CESA: Cellulose synthase A
NINV: Neutral invertases
HxK: Hexokinase
FRK: Fructokinase
CaMV 35S: Cauliflower mosaic virus 35S
WT: Wild type
GC/MS: Gas chromatography mass spectrometry
CWINV: Cell wall invertase
G1P: Glucose 1-phsophate
ph: Phloem
xy: Xylem
C: Cambium
