# Supplementary material for: MdFRK2-mediated sugar metabolism accelerates cellulose accumulation in apple and poplar
Source: Biotechnol Biofuels. 2021 Jun 15;14:137. doi: 10.1186/s13068-021-01989-9 (PMC8204578; doi:10.1186/s13068-021-01989-9)
Supplement: Supplementary file 1 — Additional file 1: Fig. S1. FRK enzyme activity in the transgenic poplars (OE#1, OE#4 and OE#9) leaves overexpressing MdFRK2. [file 13068_2021_1989_MOESM1_ESM.docx]

**Additional file 1: Fig. S1 FRK enzyme activity in the transgenic poplars (OE#1, OE#4 and OE#9) leaves overexpressing *MdFRK2*.** Bars represent the mean value ± SE (n ≥3). An asterisk indicates a significant difference at P ≤0.05.
