# Supplementary material for: MdFRK2-mediated sugar metabolism accelerates cellulose accumulation in apple and poplar
Source: Biotechnol Biofuels. 2021 Jun 15;14:137. doi: 10.1186/s13068-021-01989-9 (PMC8204578; doi:10.1186/s13068-021-01989-9)
Supplement: Supplementary file 2 — Additional file 2: Fig. S2. SUSY activity in stems of transgenic poplars (OE#1, OE#4 and OE#9) overexpressing MdFRK2. [file 13068_2021_1989_MOESM2_ESM.docx]

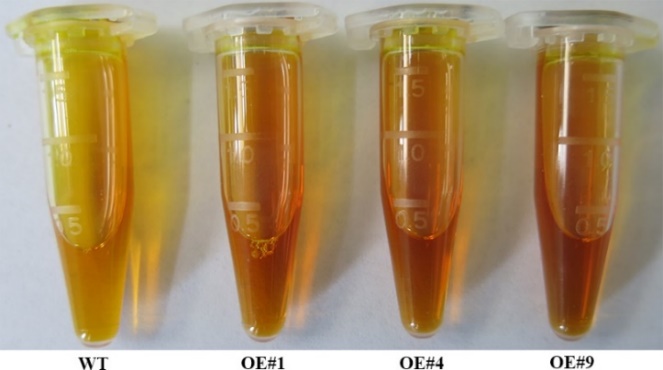


**Additional file 2: Fig. S2 SUSY activity in stems of transgenic poplars (OE#1, OE#4 and OE#9) overexpressing *MdFRK2***. SUSY, sucrose synthase
