# Supplementary material for: MdFRK2-mediated sugar metabolism accelerates cellulose accumulation in apple and poplar
Source: Biotechnol Biofuels. 2021 Jun 15;14:137. doi: 10.1186/s13068-021-01989-9 (PMC8204578; doi:10.1186/s13068-021-01989-9)
Supplement: Supplementary file 3 — Additional file 3: Table S1. Phenotypic characteristics of the transgenic poplars (OE#1, OE#4 and OE#9) overexpressing MdFRK2. [file 13068_2021_1989_MOESM3_ESM.docx]

**Additional file 3: Table S1** Phenotypic characteristics of the transgenic poplars (OE#1, OE#4 and OE#9) overexpressing *MdFRK2*

| phenotypes Index | WT | OE#1 | OE#4 | OE#9 |
| --- | --- | --- | --- | --- |
| Height (cm) | 108.25±1.82 | 108.5±1.83 | 108.25±1.75 | 108.24±1.73 |
| Stem diameter (mm) | 6.16±0.24 | 6.7±0.26 | 6.22±0.25 | 6.52±0.26 |
