# Supplementary material for: MdFRK2-mediated sugar metabolism accelerates cellulose accumulation in apple and poplar
Source: Biotechnol Biofuels. 2021 Jun 15;14:137. doi: 10.1186/s13068-021-01989-9 (PMC8204578; doi:10.1186/s13068-021-01989-9)
Supplement: Supplementary file 4 — Additional file 4: Table S2. Primers used in this study. [file 13068_2021_1989_MOESM4_ESM.docx]

**Additional file 4: Table S2** Primers used in this study

| Name/Accession no | Sequence (5’-3’) | Purpose |
| --- | --- | --- |
| qPtrCWINV2 | F: TCAACGATCCAAATGGGCCT | Quantitative expression of *PtrCWINV2* |
|  | R: GGGCTCACCATTTGGGAGAA |  |
| qPtrSUSY1 | F: ACGTGCTCTTACTCGTGTCC | Quantitative expression of *PtrSUSY1* |
|  | R: CGCCTGCCAATGTTTTCCTG |  |
| qPtrFRK1 | F: CATCAGCTGAGGCTGCTAGG | Quantitative expression of *PtrFRK1* |
|  | R: ATACCGGCAACCTTCTGACC |  |
| qPtrFRK2 | F: ACTGATCGTGAGCTTCGGTG | Quantitative expression of *PtrFRK2* |
|  | R: CGCCGACTTTCCTCCTAGTC |  |
| qPtrHxK1 | F: ATTTCCGGTCTTCGCACCTT | Quantitative expression of *PtrHxK2* |
|  | R: CAGACATGTGAGGCGTCCTT |  |
| MdActin | F: GGACAGCGAGGACATTCAGC | Real-time PCR of actin as a reference gene in apple plants |
|  | R: CTGACCCATTCCAACCATAACA |  |
| PtrActin | F: CCCATTGAGCACGGTATTGT | Real-time PCR of actin as a reference gene in poplar plants |
|  | R: TACGACCACTGGCATACAGG |  |
